# Short‐term intermittent hypoxia induces simultaneous systemic insulin resistance and higher cardiac contractility in lean mice

**DOI:** 10.14814/phy2.14738

**Published:** 2021-03-07

**Authors:** Maximin Détrait, Mélanie Pesse, Clément Calissi, Sophie Bouyon, Jacques Brocard, Guillaume Vial, Jean‐Louis Pépin, Elise Belaidi, Claire Arnaud

**Affiliations:** ^1^ University Grenoble Alpes Inserm CHU Grenoble Alpes Grenoble France; ^2^ University Lyon ENS de Lyon Inserm CNRS SFR Biosciences UCBL Lyon France

**Keywords:** cardiac function, cardiac remodeling, hyperinsulinemia, intermittent hypoxia, obstructive sleep apnea

## Abstract

**Background:**

Intermittent hypoxia (IH) is the major feature of obstructive sleep apnea syndrome, well‐known to induce cardiometabolic complications. We previously demonstrated that IH induces hyperinsulinemia and associated altered insulin signaling in adipose tissue, liver, and skeletal muscle, but impact of IH on cardiac insulin signaling and functional/structural consequences remains unknown. Therefore, the aims of this study were to investigate in both lean and obese mice the effects of chronic IH on the following: (1) cardiac insulin signaling and (2) cardiac remodeling and function.

**Methods:**

C57BL/6 J male mice were fed low‐fat (LFD) or high‐fat (HFD) diet for 20 weeks, and exposed to IH (21–5% FiO2, 60 s cycle, 8 h/day) or normoxia (N) for the last 6 weeks. Systemic insulin sensitivity was evaluated by an insulin tolerance test. Cardiac remodeling and contractile function were assessed by cardiac ultrasonography. Ultimately, hearts were withdrawn for biochemical and histological analysis.

**Results:**

In LFD mice, IH‐induced hyperinsulinemia and systemic insulin resistance that were associated with increased phosphorylations of cardiac insulin receptor and Akt on Tyr1150 and Ser473 residues, respectively. In addition, IH significantly increased cardiac interstitial fibrosis and cardiac contractility. In the HFD group, IH did not exert any additional effect, nor on insulin/Akt signaling, nor on cardiac remodeling and function.

**Conclusion:**

Our study suggests that, despite systemic insulin resistance, IH exposure mediates an adaptive cardiac response in lean but not in obese mice. Further studies are needed to investigate which specific mechanisms are involved and to determine the long‐term evolution of cardiac responses to IH.

## Graphical abstract

Despite systemic insulin resistance, intermittent hypoxia mediates an adaptive cardiac response in lean but not in obese mice. Further studies are needed to investigate which specific mechanisms are involved and to determine the long‐term evolution of cardiac responses to intermittent hypoxia.

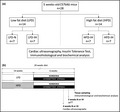

## 1 INTRODUCTION

Obstructive sleep apnea (OSA) is a common sleep disordered‐breathing that represents a major public health problem, due to its high prevalence—it affects 10–15% of the population—and its links with metabolic diseases (Drager et al., 2013). In addition, OSA is recognized as an independent risk factor for cardiovascular (hypertension, coronary diseases, heart failure) and metabolic (insulin resistance, type 2 diabetes) disorders (Levy et al., 2015). Obstructive sleep apnea is characterized by repeated episodes of partial or complete obstruction of the upper airway that leads to sleep fragmentation, intrathoracic pressure swings, and chronic intermittent hypoxia (IH), which is recognized as the major feature of OSA inducing cardiovascular and metabolic dysfunctions (Levy et al., 2015).

Experimental studies in rodents demonstrated that chronic IH, per se, is responsible for systemic insulin resistance (Drager et al., 2015) and alterations in tissue‐specific insulin signaling pathway (i.e., liver, adipose tissue and muscle) (Thomas et al., 2017). These effects of IH exposure on metabolic dysfunctions have been described in several models (i.e., lean or obese animals) and following different time‐courses of IH exposure (i.e., short‐term and long‐term) (Murphy et al., 2017; Thomas et al., 2017). Conversely, studies reporting the impact of IH on cardiac remodeling and function are more controversial. Whereas some studies demonstrated initial adaptive cardiac responses to chronic IH (Naghshin et al., 2009, 2012; Rodriguez et al., 2014), others showed that IH induces pathologic cardiac remodeling and contractile dysfunctions (Chen et al., 2005, 2008, 2010; Yin et al., 2014). Several factors could influence the pathophysiological response to chronic IH. Among them, the presence of comorbidities, such as obesity, could drive the transition from an initial adaptive state (i.e., cardiac remodeling) to a late maladaptive response (i.e., contractile dysfunction) (Yin et al., 2012).

Independently of IH, insulin resistance contributes to cardiac remodeling and contractile dysfunction. In early stages of metabolic dysfunctions, insulinemia is increased to maintain glucose homeostasis. Hyperinsulinemia results in “sustained” myocardial IRS1 and Akt phosophorylation that contributes to left ventricular remodeling. Later, and despite persistent hyperinsulinemia, glucose homeostasis is no longer regulated and cardiac insulin resistance occurs, participating in cardiac contractile dysfunctions (Riehle & Abel, 2016). This deleterious impact of hyperinsulinemia on cardiac remodeling and contractile dysfunction is well‐described in animal models of obesity‐induced insulin resistance (Riehle & Abel, 2016) and has also been described to exacerbate systolic dysfunction induced by pressure overload (Shimizu et al., 2010).

Currently, we demonstrated previously that IH induces hyperinsulinemia and associated altered insulin signaling in adipose tissue, liver, and skeletal muscle (Thomas et al., 2017), but the impact of IH‐induced insulin resistance on cardiac insulin signaling and functional/structural cardiac consequences remains unknown. Therefore, the aims of this study were to investigate, in both lean and obese mice, the effects of chronic IH on the following: (1) cardiac insulin signaling and (2) cardiac remodeling and function.

## 2 MATERIAL AND METHODS

### 2.1 Ethical approval

All experiments were performed in accordance with the Guide for the Care and Use of Laboratory Animals (National Instituted of Health publication 85‐23, revised 1996) and have received approval from the ethical committee of the Université Grenoble Alpes and the “Ministère de l'enseignement supérieur et de la recherche” (no. 2256.02).

### 2.2 Animals and experimental design

**FIGURE 1:**
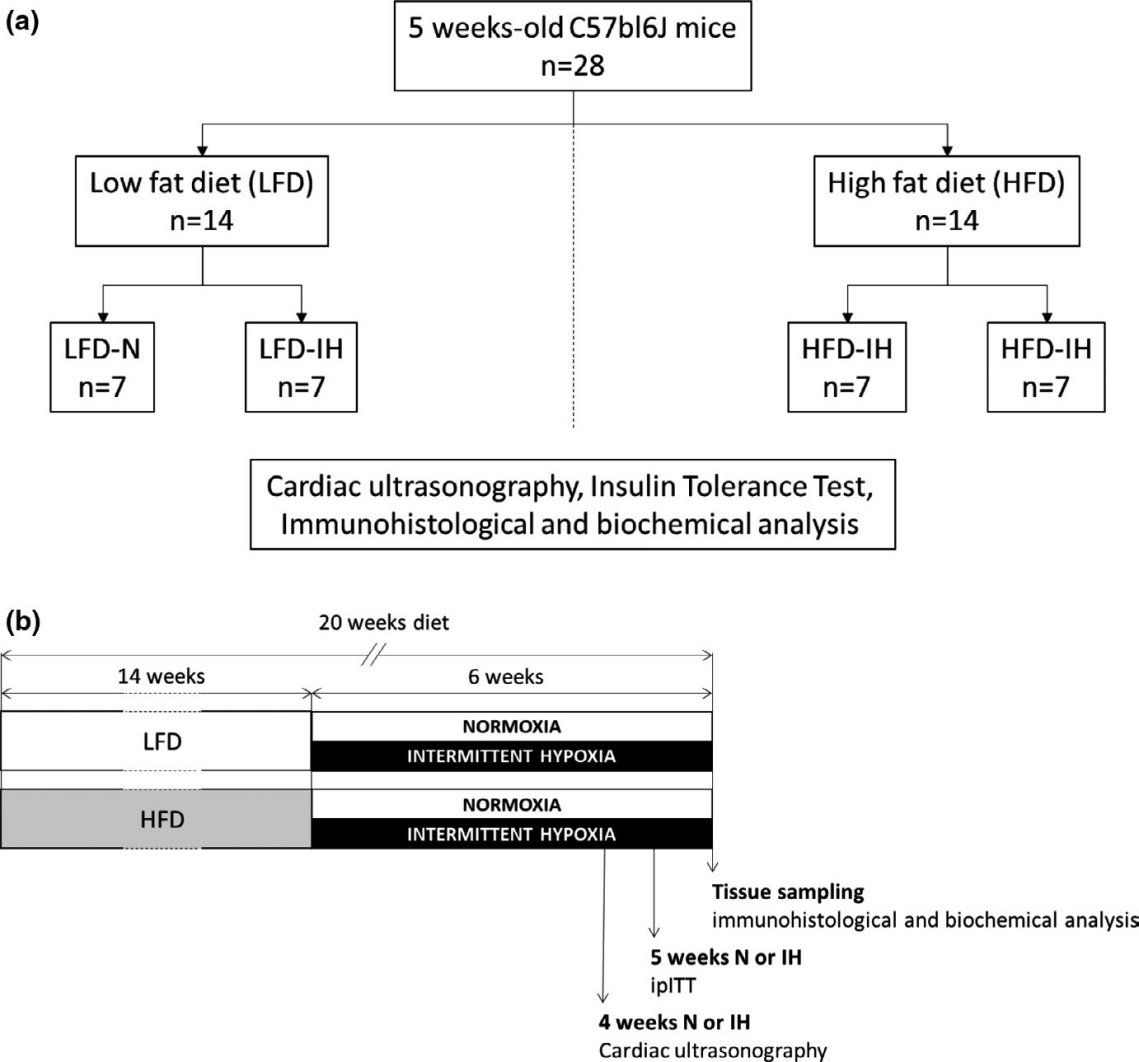
Experimental groups (a) and study design (b)

Twenty‐eight 5‐week‐old male C57BL/6 J mice (Janvier) were housed under standard conditions in conventional cages with *ad libitum* access to water and food. Mice were randomized into two groups fed either a control low‐fat (LFD) diet (10 kcal% Fat, catalogue no. D12450 J, Research Diets, New Brunswick, USA) or a high fat (HFD) diet (60 kcal% Fat, catalogue no. D12492, 60% fat, Research Diets) for 20 weeks (*n* = 14 per group). During the last 6 weeks of the protocol, each group of mice was randomized into two subgroups (*n* = 7 per group), exposed to normoxia (N) or chronic intermittent hypoxia (IH) (Figure 1(a)). The animals were exposed in their housing cages to 60 s IH cycles (inspired oxygen fraction oscillating from 21% to 5% every 30 s), for 8 h during sleeping time (6 am–2 pm). In parallel, control animals were exposed to similar air–air cycles (21% O_2_), in order to reproduce equivalent levels of noise and air turbulences related to gas circulation as IH, without hypoxia. At 4 weeks exposure, mice were submitted to cardiac ultrasonography; at 5 weeks, fasting glycemia and insulinemia were measured and an intraperitoneal (i.p) insulin tolerance test (ipITT) was performed; tissue sampling was realized at 6 weeks. Experimental design is summarized in Figure 1(b).

### 2.3 Insulin tolerance test

The week before mice euthanasia, an intraperitoneal (i.p) insulin tolerance test (ipITT) was performed by a training technician, to assess whole‐body insulin sensitivity. Briefly, mice were fasted for 6 h and insulin was administrated (0.5 U/kg total body weight, i.p). Blood samples were drawn from the tail vein of conscious mice at different time points (0–15–30–45–60 and 90 min) to determine blood glucose concentrations using a glucometer (One Touch VERIO). Fasting insulinemia was measured using a commercial insulin kit (Millipore).

### 2.4 Cardiac ultrasonography

Cardiac ultrasonography was performed by a trained technician during the fourth week of exposure, using a Vevo 2100 imaging system from Visual Sonics with a 22–55 MHz MS550D transducer. Mice were anaesthetized with isofluorane (1%) supplemented with O_2_. Body temperature, respiratory rate and ECG were monitored and heart rate was maintained at 350–400 beats/min during the measurements of cardiac parameters. Systolic function parameters, including the ejection fraction (EF) and fractional shortening (FS) were measured by two‐dimensional parasternal short‐axis imaging plane of M‐mode traces close to the papillary muscle level.

### 2.5 Heart sampling

At the end of IH or N exposure, mice were fasted for 6 h, then euthanized by cervical dislocation, after an insulin (0.5 U/kg total body weight, i.p) or NaCl (100 µl/100 g body weight, i.p) injection (*n* = 3 and 4 for NaCl and insulin administration respectively). Fifteen minutes later, hearts were rapidly sampled and weighted. The mid‐ventricular part of heart was fixed in paraformaldehyde (PFA) for histology study. Apex and bases of hearts were quickly frozen, for protein biology analysis.

### 2.6 Western blot analysis

Snap‐frozen hearts were homogenized and lysed using ceramic beads in Precellys 24 (5000 rpm, 2 × 10 s‐5 s) to extract nuclear and cytosolic proteins (Nuclear Extract protocol, Active Motif). Protein concentration was calculated using a Bradford assay (Bradford reagent, Sigma‐Aldrich). Cytosolic proteins (30 µg) were separated by 8–10% SDS polyacrylamide gels and transferred to polyvinylidene difluoride membranes. Next, membranes were blocked for 1 h at room temperature with 5% nonfat milk in TRIS‐buffered saline (TBS) with Tween 20 (0.1%). Membranes were then incubated overnight at 4°C with the following primary antibodies in TBS‐Tween 20–5% nonfat milk: ^Ser473^phospho‐Akt, ^Tyr1150^phospho‐IRβ, Akt1 (1:1000, Millipore), Irβ (1:1000, Santa Cruz Biotechnology), and α‐tubulin (1:2000, Santa Cruz Biotechnology). Membranes were then incubated for 1 h at room temperature with the appropriate horseradish peroxidase‐conjugated anti‐IgG (1:5000, Jackson ImmunoResearch). Proteins were visualized by enhanced chemiluminescence with the Western Blot ECL substrate (Clarity, Bio‐Rad) and video acquisition (chemidoc‐xrs‐system, Bio‐ Rad). Relative amount of protein was quantified by densitometry (Image Lab, Bio‐Rad) and expressed as a ratio of the appropriate loading control. Phosphorylated proteins were expressed relative to total protein, and nonphosphorylated proteins were expressed relative to tubulin. Finally, results were expressed as the phosphorylated‐to‐total ratio relative to LFD‐NaCl or HFD‐NaCl groups.

### 2.7 RT‐qPCR

Total mRNA was extracted from snpap‐frozen heart using TRI reagent (Sigma‐Aldrich) according to the manufacturer's specifications. Total RNA (0.1 µg) was reversely transcribed to cDNA using iScript Reverse Transcription Supermix (C‐1000 Thermal Cycler, Bio‐Rad). Quantitative real‐time PCR was then performed using SsoAdvanced Universal SYBR Green Supermix (Bio‐Rad) and PCR primers (Sigma‐Aldrich). mRNA expression was normalized to Actin mRNA levels and expressed as fold change compared to Normoxic mice using 2^ΔΔCt^ method (where C_t_ is threshold cycle). Primers are listed in Table S1.

### 2.8 Histology

Mouse heart tissue was fixed in PFA then embedded in paraffin and serially sectioned at 5 µm on a microtome. Tissue sections were stained with Sirius red to assess interstitial fibrosis. Briefly, paraffin‐embedded sections were deparaffinized and washed in distilled water. Slides were then stained for 1 h with Sirius red (0.1% of Sirius red in saturated aqueous picric acid) and washed twice in acetic acid solution (0.5%). Slides were rinsed using absolute alcohol, dehydrated, cleared, and then mounted for observation. Images were acquired using axioscan (Zeiss, Göttingen, Germany, X20). Three mice per group were used and 10 to 12 images per mice were analyzed in left and right ventricles and septum by a blinded experimenter. Briefly, interstitial fibrosis was automatically segmented from these images, based on its specific reddish hue, with the help of a home‐made ImageJ macro (https://github.com/jbrocardplatim/Cardiac‐Fibrosis, code available in Supplementary data). Percentages of fibrosis were then calculated as the area of fibrosis divided by the area of the stained tissue. In order to determine cardiomyocytes cross‐sectional area, tissue sections were stained with Wheat germ agglutinin (WGA) Oregon Green 488 conjugate (Molecular Probes). Staining was performed following standard procedures and images were acquired using the confocal microscopy (LSM5 10 Meta confocal microscope; Zeiss, Oberkochen, Germany). Cross‐sectional area analysis (three mice per group, 10 to 15 images per mice and 50 cardiomyocytes per image) was performed by a blinded experimenter using Image J (*Stochastic watershed* plugin, adapted by Dr A. Fertin, TIMC‐IMAG‐UMR5525, France).

### 2.9 Statistical analysis

All data are expressed as mean ± SEM. For each comparison group, normality and equal variance were tested using Shapiro–Wilk and Bartlett tests, respectively. When these assumptions were met, results were analyzed using Student t‐tests or two‐way ANOVA, followed by post hoc Tuckey's multiple comparison tests. In other conditions and when sample size was too small, Kruskal–Wallis test was used, followed by Dunn's multiple comparison tests (GraphPad Prism Software). Differences between groups were considered statistically significant at *p* < 0.05.

## 3 RESULTS

### 3.1 Impact of high‐fat diet on metabolic and cardiac parameters

**FIGURE 2:**
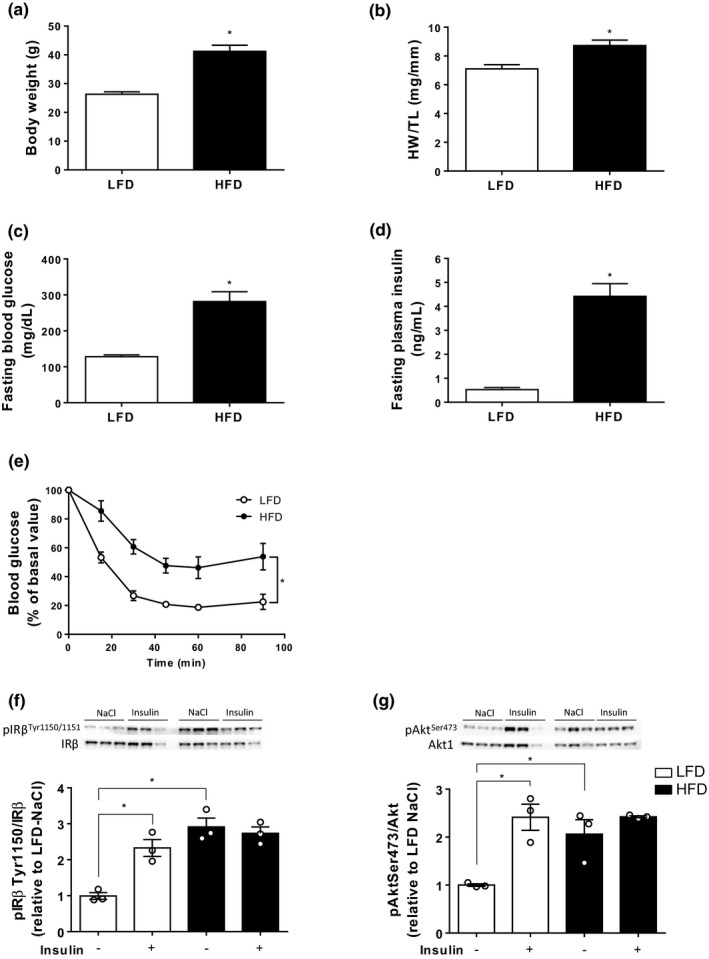
Impact of 20 weeks high‐fat diet (HFD) on body weight (a) and heart weight‐to‐tibia length ratio (HW/TL) (b). Fasted blood glucose (c) and plasma insulin (d) levels were monitored after 19 weeks diet and insulin tolerance test was performed (e). Data are expressed as means ± SEM (*n* = 7). The protein expression of IRβ, Akt, and phosphorylation states on ^Tyr1150I^Rβ and ^Ser473^Akt was determined by Western blot (f and g) and quantified by densitometric analysis. The phospho‐to‐total ratios were calculated and expressed as fold change relative to LFD control mice. Data are expressed as means ± SEM (*n* = 3). *p < 0.05 compared to LFD

HFD significantly increased body weight (Figure 2(a)), fasting blood glucose (Figure 2(c)), and plasma insulin levels (Figure 2(d)). This was associated with a global reduction in systemic insulin response during ipITT (Figure 2(e)). Concomitantly, under basal conditions, HFD significantly increased basal phosphorylations of insulin receptor β and Akt on Tyr1150 (Figure 2(f)) and Ser473 (Figure 2(g)), respectively. In addition, whereas IRβ and Akt phosphorylations were increased in the LFD group, insulin administration did not increase IRβ and Akt phosphorylation states in the HFD group (Figure 2(f) and (g)). Regarding cardiac remodeling and function, HFD significantly increased heart weight‐to‐tibia length ratio (Figure 2(b)), interventricular septum thickness, without any significant effect on ejection fraction and fractional shortening (Table S2).

### 3.2 Cardiometabolic impact of chronic intermittent hypoxia in LFD mice

**FIGURE 3:**
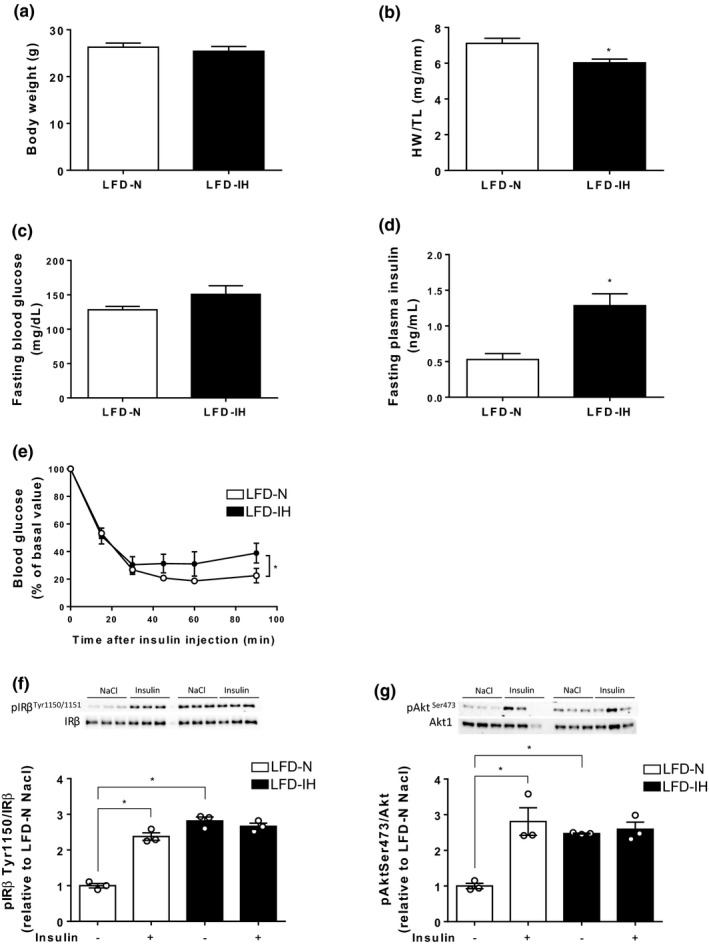
Impact of 6 weeks IH on metabolic parameters in LFD mice. Body weight (a) and heart weight‐to‐tibia length ratio (HW/TL) (b) were monitored after 20 weeks diet and 6 weeks IH exposure. Fasted blood glucose (c) and plasma insulin (d) levels were monitored after 19 weeks LFD and insulin tolerance test was performed (e). Data are expressed as means ± SEM (*n* = 7). The protein expression of IRβ, Akt and phosphorylation states on ^Tyr1150^IRβ and ^Ser473^Akt were determined under basal conditions or 15 min after insulin injection by Western blot (f and g) and quantified by densitometric analysis. The phospho‐to‐total ratios were calculated and expressed as fold change relative to LFD‐N‐NaCl condition. Data are expressed as means ± SEM (*n* = 3). *p < 0.05, compared to LFD‐N

**FIGURE 4:**
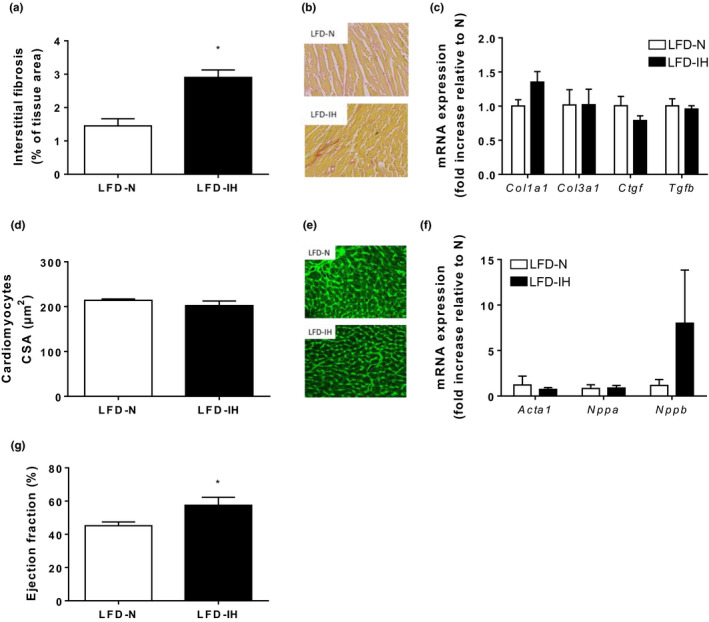
Impact of 6 weeks IH on cardiac remodeling and function in LFD mice. Quantification of cardiac interstitial fibrosis expressed as percentage of tissue area (10–12 images analyzed per mice; 3 mice per group) (a). Representative images of interstitial fibrosis in N and IH mice (b). mRNA expression of fibrosis markers *Col1a1*, *Col3a1*, *Ctgf* and *Tgfβ* (*n* = 3) (c). Cardiomyocyte cross‐sectional (CSA) area quantification (around 500 cardiomyocytes analyzed; 3 mice per group) (d) and representative WGA stainings (e). Quantification of mRNA expression of markers of cardiac hypertrophy, *Acta1*, *Nppa*, and *Nppb* (*n* = 3) (f). Echocardiographic assessment of ejection fraction in LFD mice (*n* = 7) (g). Data are expressed as means ± SEM, *p < 0.05

In the LFD mice, IH had no effect on final body weight (Figure 3(a)). IH did not change fasting glucose levels (Figure 3(c)) but significantly increased plasma insulin levels (Figure 3(d)). To further investigate the effect of IH on systemic response to insulin, mice were subjected to an insulin tolerance test in vivo. IH mice exhibited an impaired whole‐body insulin sensitivity compared to normoxic mice (Figure 3(e)). Insulin‐induced increased in Tyr1150IRβ and Ser473Akt phosphorylations observed in normoxic mice was blunted in hypoxic ones (Figure 3(f) and (g)). Of note, IH induced an increase in IRβ and Akt phosphorylations in basal conditions (Figure 3(f) and (g)), suggesting that IH, *per se*, upregulates cardiac insulin signaling. Regarding cardiac impact, IH induced interstitial fibrosis, as shown by Sirius red staining (Figure 4(a) and (b)) that was associated with a trend to increase in *Col1a1* mRNA expression (Figure 4(c)). Cardiomyocytes cross‐sectional areas, assessed through Wheat germ agglutinin (WGA) staining, were not different between LFD‐N and LFD‐IH groups (Figure 4(d) and (e)) and we did not observe any re‐expression of the fetal gene program (Figure 4(f)). Finally, in the LFD mice, IH induced a reduction in left ventricular diameter (LVDs) (2.654 ± 0.214 vs 3.161 ± 0.099* in IH vs. N respectively, *p < 0.05) (Table 1). This was associated with a significant increase in ejection fraction (57.5 ± 4.80 vs. 45.2 ± 2.2%* in IH vs. N, respectively, *p < 0.05) and fractional shortening (26.8 ± 1.1 vs. 22.2 ± 1.3% in IH vs. N respectively, *p < 0.05) (Table 1 and Figure 4(g)).

**TABLE 1:** Echocardiographic parameters assessed in LFD mice

	LFD‐N	LFD‐IH
HR (beats/min)	357 ± 15	372 ± 21
IVSd (mm)	0.688 ± 0.022	0.744 ± 0.020
IVSs (mm)	0.941 ± 0.025	1.021 ± 0.056
LVDd (mm)	4.062 ± 0.083	3.785 ± 0.134
LVDs (mm)	3.161 ± 0.099	2.654 ± 0.214*
LVPWd (mm)	0.722 ± 0.022	0.692 ± 0.021
LVPWs (mm)	0.929 ± 0.017	0.983 ± 0.051
EF (%)	45.2 ± 2.2	57.5 ± 4.8*
FS (%)	22.2 ± 1.3	26.8 ± 1.1

Results are expressed as means ± SEM (*n* = 7 per group).

Abbreviations: EF, ejection fraction; FS, fractional shortening; HR, heart rate; IH, intermittent hypoxia; IVSd, diastolic interventricular septum; IVSs, systolic interventricular septum; LFD, low‐fat diet; LVDd, diastolic left ventricular diameter; LVDs, systolic left ventricular diameter; LVPWd, diastolic left ventricular posterior wall; LVPWs, systolic left ventricular posterior wall; N, normoxia.

* *p* < 0.05, compared to LFD‐N mice.

### 3.3 Cardiometabolic impact of chronic intermittent hypoxia in HFD mice

**FIGURE 5:**
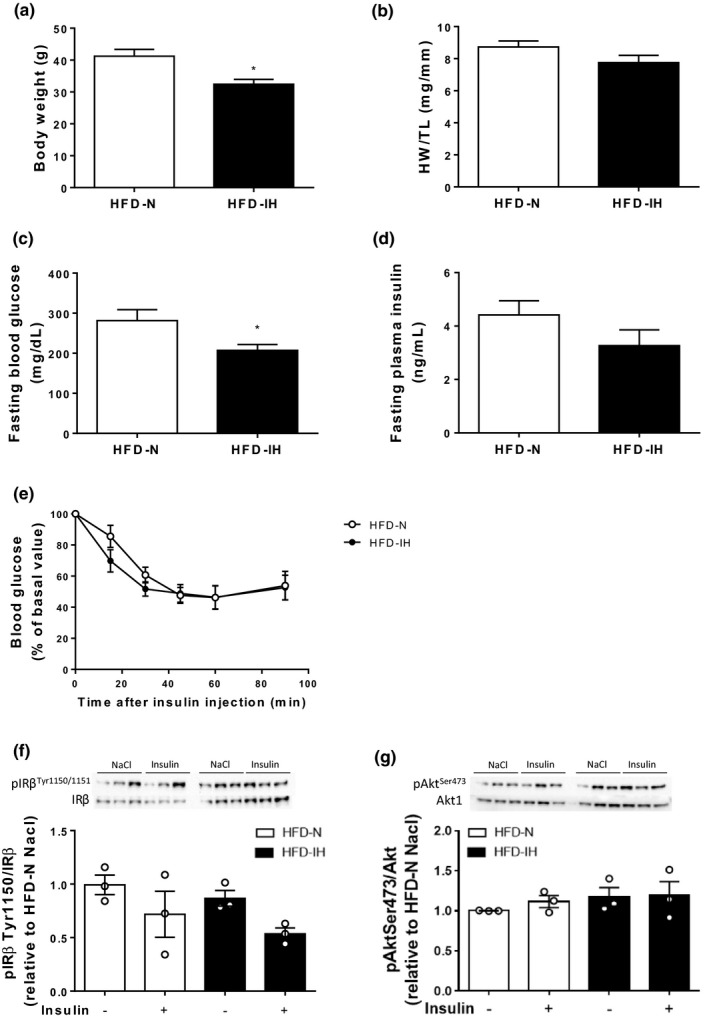
Impact of 6 weeks IH on metabolic parameters in HFD mice. Body weight (a) and heart weight‐to‐tibia length ratio (HW/TL) (b) were monitored after 20 weeks diet and 6 weeks IH exposure. Fasted blood glucose (c) and plasma insulin (d) levels were monitored after 19 weeks HFD and insulin tolerance test was performed (e). Data are expressed as means ± SEM (*n* = 7). The protein expression of IRβ, Akt and phosphorylation states on ^Tyr1150^IRβ and ^Ser473^Akt were determined under basal conditions or 15 min after insulin injection by Western blot (f and g) and quantified by densitometric analysis. The phospho‐to‐total ratios were calculated and expressed as fold change relative to HFD‐N‐NaCl condition. Data are expressed as means ± SEM (*n* = 3). *p < 0.05 compared to HFD‐N

**FIGURE 6:**
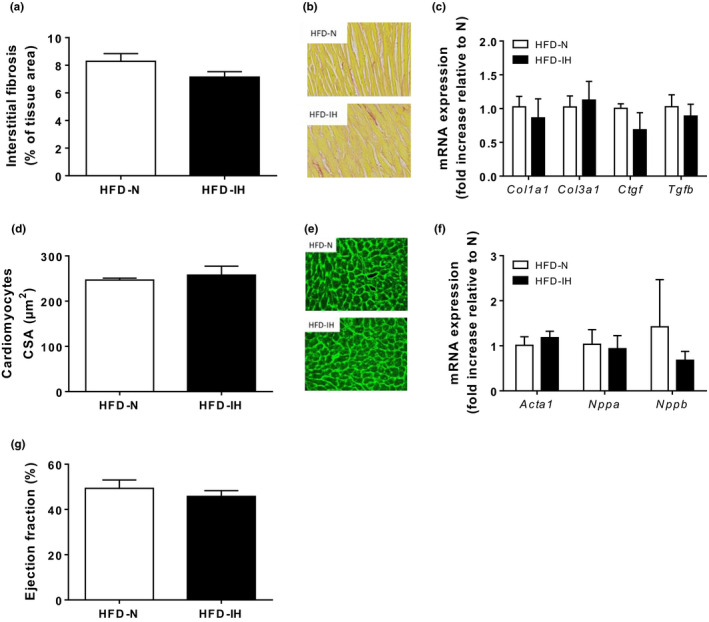
Impact of 6 weeks IH on cardiac remodeling and function in HFD mice. Quantification of cardiac interstitial fibrosis expressed as percentage of tissue area (10–12 images analyzed per mice; 3 mice per group) (a). Representative images of interstitial fibrosis in N and IH mice (b). mRNA expression of fibrosis markers *Col1a1*, *Col3a1*, *Ctgf* and *Tgfβ* (*n* = 3) (c). Cardiomyocyte cross‐sectional area (CSA) quantification (around 500 cardiomyocytes analyzed; 3 mice per group) (d) and representative WGA stainings (e). Quantification of mRNA expression of markers of cardiac hypertrophy, *Acta1*, *Nppa*, and *Nppb* (*n* = 3) (f). Echocardiographic assessment of ejection fraction in HFD mice (*n* = 7) (g). Data are expressed as means ± SEM

We first validated that HFD mice induced metabolic disorders (Table S1 and Figure 2). Then, we evaluated whether IH could exert additive effects in a model of diet‐induced obesity. In HFD conditions, IH reduced body weight (Figure 5(a)) and fasting blood glucose levels (Figure 5(c)). We did not observe any effect of IH on fasting plasma insulin (Figure 5(d)), nor on the dynamic response to insulin (ITT) (Figure 5(e)), nor on IRβ and Akt phosphorylation states in basal and post‐insulin injection situations (Figure 5(f) and (g)). In the same manner, we did not observe any changes in heart weight‐to‐tibia length ratio (Figure 5(b)), cardiac fibrosis and cardiomyocytes size between HFD‐N and HFD‐IH mice (Figure 6(a)–(f)). We only observed a significantly decrease in interventricular septum thickness in HFD‐IH compared to HFD‐N (Table 2), that was not associated with changes in ejection fraction (45.8 ± 2.5 vs. 49.4 ± 3.7% in IH vs. N, respectively) and fractional shortening (22.6 ± 1.5 vs. 24.9 ± 2.3% in IH vs. N, respectively) (Table 2 and Figure 6(g)).

**TABLE 2:** Echocardiographic parameters assessed in HFD mice

	HFD‐N	HFD‐IH
HR (beats/min)	355 ± 14	337 ± 12
IVSd (mm)	0.868 ± 0.028	0.716 ± 0.027*
IVSs (mm)	1.182 ± 0.038	0.980 ± 0.034*
LVDd (mm)	4.184 ± 0.173	4.121 ± 0.101
LVDs (mm)	3.157 ± 0.203	3.193 ± 0.123
LVPWd (mm)	0.843 ± 0.035	0.777 ± 0.020
LVPWs (mm)	1.099 ± 0.061	0.980 ± 0.042
EF (%)	49.4 ± 3.7	45.8 ± 2.5
FS (%)	24.9 ± 2.3	22.6 ± 1.5

Results are expressed as means ± SEM (*n* = 7 per group).

Abbreviations: EF, ejection fraction; FS, fractional shortening; HFD, high‐fat diet; HR, heart rate; IH, intermittent hypoxia; IVSd, diastolic interventricular septum; IVSs, systolic interventricular septum; LVDd, diastolic left ventricular diameter; LVDs, systolic left ventricular diameter; LVPWd, diastolic left ventricular posterior wall; LVPWs, systolic left ventricular posterior wall; N, normoxia.

* *p* < 0.05, compared to HFD‐N mice.

## 4 DISCUSSION

Exposure to IH induces systemic insulin resistance and alters the insulin signaling pathway in the main metabolic organs (i.e., adipose tissue, liver, and skeletal muscle); however, the cardiac impact of this IH‐induced insulin resistance remains unclear. In this study, we demonstrated that, in lean mice, 6 weeks exposure to IH is responsible for hyperinsulinemia, systemic insulin resistance, cardiac insulin signaling modifications, cardiac interstitial fibrosis, and increased cardiac contractility. Interestingly, in diet‐induced obese mice already exhibiting basal hyperinsulinemia and systemic insulin resistance, IH did not exert additional systemic metabolic alterations, nor on cardiac insulin signaling, nor on cardiac remodeling and function.

### 4.1 Cardiometabolic response to chronic IH in lean mice

According to the literature (Drager et al., 2015; Muhl & Pfeilschifter, 2003; Murphy et al., 2017; Thomas et al., 2017) and although body weight and fasting blood glucose were similar to normoxic mice, low‐fat diet mice exposed to chronic IH exhibited common features of systemic insulin resistance, such as increased fasting plasma insulin levels and impaired insulin sensitivity during ITT. Phosphorylation states of both insulin receptor and Akt were unexpectedly upregulated after 6 weeks IH and the response to exogenous insulin stimulation was blunted compared to normoxic mice. Thus, in myocardium, the impact of IH on insulin‐signaling seems to partially differ from those we previously obtained in the main insulin‐targeted tissues (i.e., WAT, skeletal muscle, and liver) showing that IH alters insulin‐signaling pathway by lowering the insulin‐induced phosphorylation of IR and downstream targets, with no modifications in basal conditions (Murphy et al., 2017; Thomas et al., 2017). These results suggest a specific organ‐dependent metabolic response to IH and the underlying molecular mechanisms remain to be elucidated.

In cardiac tissue, such hyperactivation of insulin/Akt signaling pathway has been described in other pathophysiological contexts, such as early stages of heart failure (i.e., pressure overload model) (Shimizu et al., 2010) or metabolic syndrome (i.e., ob/ob mice) (Cook et al., 2010). In these two models, insulin/Akt signaling hyperactivation results, in part, from compensatory hyperinsulinemia and promotes early adaptive cardiac response and a metabolic switch from fatty acid oxidation to glycolytic way (Riehle & Abel, 2016). However, persistent and excessive signaling is associated with adverse LV remodeling (hypertrophy and/or cardiac fibrosis) and subsequent cardiac dysfunction (Riehle & Abel, 2016).

In this study, we further demonstrated an increased cardiac contractility in the IH group, suggesting as well a compensatory cardiac response to 4 weeks IH in lean mice. This was associated with increased cardiac interstitial fibrosis, but no cardiac hypertrophy. These results are in accordance with previous studies demonstrating that 4 weeks IH increased cardiac contractility, independently of cardiac hypertrophy (Naghshin et al., 2009, 2012; Rodriguez et al., 2014), whereas other studies demonstrated cardiac remodeling (i.e. apoptosis, interstitial fibrosis, hypertrophy) and contractile dysfunction (i.e., reduction in ejection fraction) (Chen et al., 2008, 2010) upon IH exposure. These discrepancies could be explained by several experimental parameters such as rodent species, genetic background, as well as hypoxia patterns (depth, number of cycles, duration). Taken together, these results also suggest that the cardiac response to IH could be biphasic, with an early compensatory response, followed by a maladaptive remodeling and subsequent functional failure.

Several mechanisms could be involved in these cardiometabolic responses to IH. As described earlier, hyperinsulinemia contributes to early increased cardiac contractility (Riehle & Abel, 2016); previous studies also underlined the contributing role of IH‐induced sympathetic activation (Bourdier et al., 2016; Morand et al., 2018; Tamisier et al., 2011), as beta‐blockers prevent the IH‐induced increased cardiac contractility (Naghshin et al., 2009). In addition, as hyperinsulinemia, which is well‐known to compensate hyperglycemia in the early phase of metabolic disease (Riehle & Abel, 2016), sympathetic activation induces short‐term adaptive response to stress that becomes maladaptive when activation is sustained and persistent, ultimately contributing to cardiac pathological remodeling and contractile dysfunction (El‐Armouche & Eschenhagen, 2009). Taken together, this suggests that, in early stages of IH exposure, both hyperinsulinemia and sympathetic activation could represent adaptive mechanisms that, with prolonged exposure or in presence of comorbidities might ultimately induce pathological remodeling and contractile dysfunction. Further studies are needed to dissect the specific interlinked mechanisms of IH‐induced hyperinsulinemia and sympathetic activation in order to better understand and prevent the maladaptive phase of long‐term IH exposure.

### 4.2 Cardiometabolic response to chronic IH in diet‐induced obese mice

Obesity is the most prevalent comorbidities of OSA (Levy et al., 2015) and could exert synergistic effects to IH on myocardium (Yin et al., 2012). As currently described in the literature, our experimental model of diet‐induced obesity led to systemic insulin resistance with increased levels of fasting blood glucose and insulin, and a systemic altered response to insulin compared to mice fed with low‐fat diet. In the same manner than in lean mice exposed to IH, we showed that high fat diet upregulated cardiac insulin/Akt signaling in the basal state that could result from hyperinsulinemia, whereas response to insulin stimulation was blunted, suggesting an insulin resistance state (Cook et al., 2010; Riehle & Abel, 2016). These mice also exhibited cardiac hypertrophy that was not associated with contractile dysfunction. Interestingly, IH did not exert any additive effect, nor on systemic metabolic parameters (i.e., no additional increase in insulinemia), nor on cardiac insulin/Akt signaling, cardiac remodeling and function in these high‐fat diet mice. This is in accordance with our previous study demonstrating that IH exerts synergistic effect with obesity on insulin resistance only after body weight adjustment, which was not performed in this study due to the small number of animals (Murphy et al., 2017). In addition, contrary to lean mice, IH did not increase cardiac contractility in obese mice, which could traduce that obese mice cannot develop an adaptive cardiac response to IH. Same results were reported by Rodriguez et al. who demonstrated that ob/ob mice failed to develop a compensatory contractile response to IH (Rodriguez et al., 2014). Besides, in this study, the authors demonstrated that obese mice were unable to increase glycolytic rate, which was proposed as an adaptive mechanism in lean mice (Rodriguez et al., 2014). Finally, while we proposed that sympathetic activation could contribute to the adaptive contractile response to IH, previous study showed that high fat diet impaired cardiac beta‐adrenergic signaling in early stages of diabetes without any effect on global cardiac function (Fu et al., 2017). This altered beta‐adrenergic signaling might explain, at least in part, the inability of obese mice to increase cardiac contractility to IH.

### 4.3 Novelty of the study and perspectives

As stated earlier in the discussion, previous studies demonstrated independently the impact of IH on systemic metabolic disorders or cardiac function in isolation. To our knowledge, our study is the first to describe a simultaneous effect of IH on systemic metabolism and cardiac function, with different responses observed in lean versus obese mice. In addition, whereas many studies reported the impact of IH on insulin signaling in liver, adipose tissue or skeletal muscle, our study assessed insulin/akt signaling in myocardium, which has not been reported yet.

However, one of the major weakness of this study is that our experimental design does not allow to conclude on a direct mechanistic link between systemic metabolic disturbances, cardiac insulin signaling, and cardiac remodeling and function. We demonstrated that IH‐induced systemic metabolic troubles occur simultaneously with increased myocardial contractility in lean mice, which strongly suggests that, despite metabolic disturbances, short‐term IH exposure induced early adaptive cardiac response in lean mice. As we cannot observe such increase in cardiac function in obese mice, we speculate that metabolic disorders induced by HFD blunt the IH‐induced cardiac adaptive response at 4 weeks, which could result in a quicker cardiac decompensation after longer IH exposure. Further studies are mandatory to support this hypothesis and to clearly demonstrate the link between IH‐induced systemic metabolic troubles, cardiac insulin signaling, and cardiac remodeling and function along the time, in both lean and obese mice. The ultimate aim will be to decipher specific mechanisms, depending on the metabolic status, in order to propose optimized individualized preventive strategies in apneic patients.

## 5 CONCLUSION

The combination of our present data and the literature suggest that, in early stages of IH exposure, hyperinsulinemia and beta‐adrenergic signaling could jointly mediate an adaptive/compensatory cardiac response in lean mice. Deciphering specific interconnected mechanisms seems mandatory to identify specific target and prevent long‐term IH‐induced pathological remodeling and contractile dysfunction. The discrepancy we observed between lean and obese mice also deserve to be investigated in detail, since specific mechanisms could explain variable cardio‐metabolic responses in apneic patients exhibiting different OSA phenotype and/or associated co‐morbidities.

## CONFLICT OF INTEREST

None.

## Supporting information



Table S1‐S2

Supplementary data

## Data Availability

The data, methods used in the analysis, and materials used to conduct the study are available from the corresponding author upon reasonable request.
