# Supplementary figures and images for: Short‐term intermittent hypoxia induces simultaneous systemic insulin resistance and higher cardiac contractility in lean mice
Source: Physiol Rep. 2021 Mar 7;9(5):e14738. doi: 10.14814/phy2.14738 (PMC7937943; doi:10.14814/phy2.14738)

## Slide 1
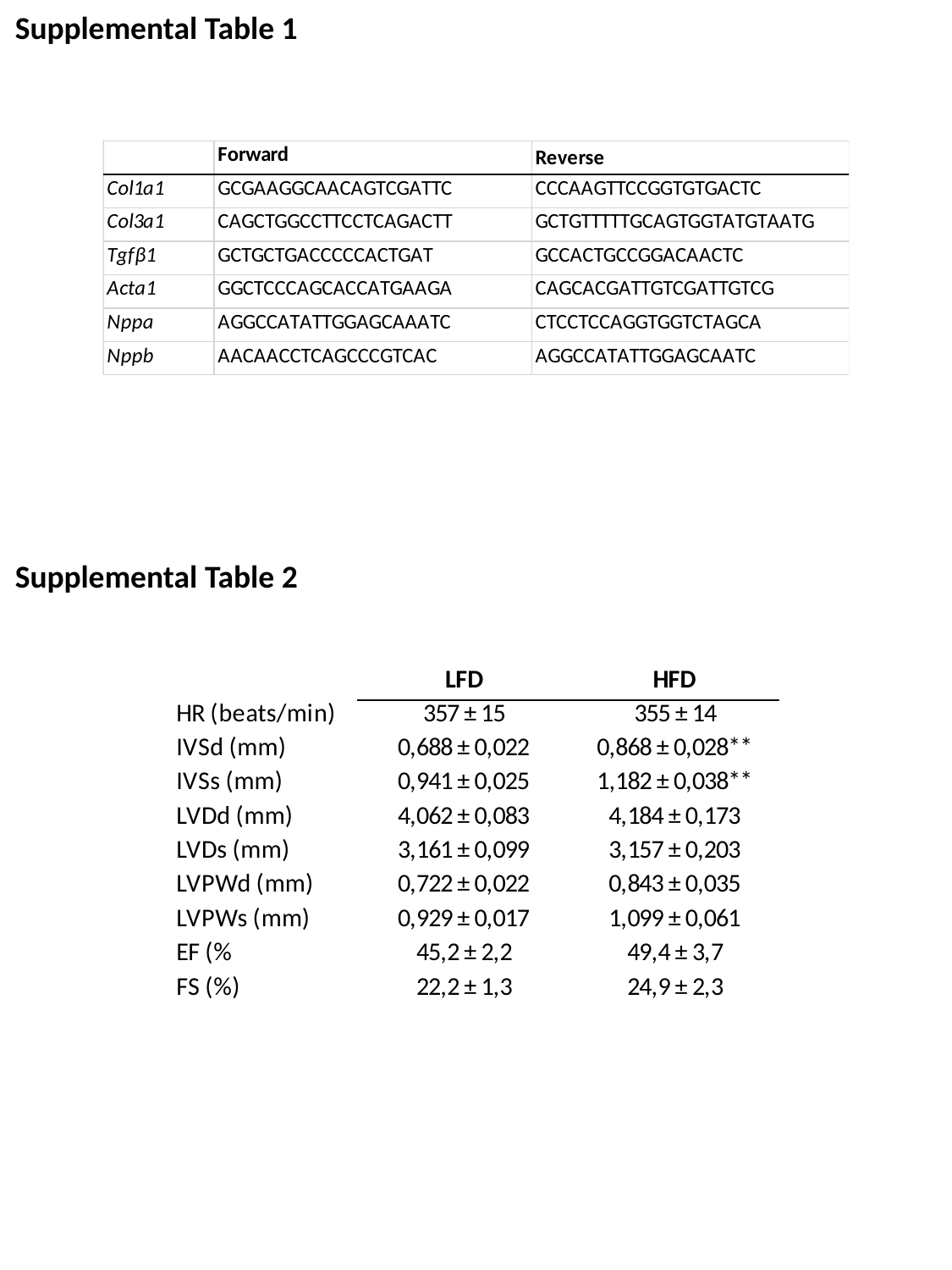

Supplemental Table 1
Supplemental Table 2

Supplement: Supplementary file 1 — Table S1‐S2 [file PHY2-9-e14738-s001.pptx]
