# Supplementary material for: Short‐term intermittent hypoxia induces simultaneous systemic insulin resistance and higher cardiac contractility in lean mice
Source: Physiol Rep. 2021 Mar 7;9(5):e14738. doi: 10.14814/phy2.14738 (PMC7937943; doi:10.14814/phy2.14738)
Supplement: Supplementary file 2 — Supplementary data [file PHY2-9-e14738-s002.docx]

**Online supplemental material and data**

**Quantification of cardiac interstitial fibrosis**

Code for ImageJ macro (<https://github.com/jbrocardplatim/Cardiac-Fibrosis>)

//Automatic quantification of fibrosis in Sirius red-stained heart sections imaged with an

//AxioScan.Z1 * Claire Arnaud, HP2 @Jacques Brocard, 2019

run("Set Measurements...", "area mean standard redirect=None decimal=3");getPixelSize(unit, pw, ph, pd);

if (unit=="inches"){

run("Properties...", "channels=1 slices=1 frames=1 unit=microns pixel_width=0.22 pixel_height=0.22 voxel_depth=1");

run("Save");

getPixelSize(unit, pw, ph, pd);

}

titre=getTitle();

dir=getDirectory("image");

titre=substring(titre,0,lengthOf(titre)-4);

//Splitting and smoothing of the original images

run("Duplicate...", "title=image");

run("Split Channels");

selectWindow("image (blue)");

run("Smooth");

selectWindow("image (green)");

run("Smooth");

selectWindow("image (red)");

run("Smooth");

imageCalculator("Add create 32-bit", "image (green)","image (blue)");

selectWindow("Result of image (green)");

//Production of a dark image of the tissue

resetMinAndMax();

run("Enhance Contrast", "saturated=0.35");

setOption("ScaleConversions", true);

run("8-bit");

//Image data harvesting and subsequent adaptation of the threshold

run("Set Measurements...", "area mean standard limit redirect=None decimal=3");

run("Select All");

run("Measure");

moy=getResult("Mean",0);

sd=getResult("StdDev",0);

setAutoThreshold("Default");

setThreshold(0, moy+sd-10);

//setThreshold(0, 200);

setOption("BlackBackground", true);

run("Convert to Mask");

tis=titre+"_tissue.tif";

saveAs("Tiff", dir+tis);

//Production of an image = green/(red+blue) -> fibrosis

imageCalculator("Add create 32-bit", "image (blue)","image (red)");

rename("BG");

imageCalculator("Divide create 32-bit", "image (green)","BG");

rename("FIB");

run("8-bit");

run("Invert");

run("Duplicate...", " ");

run("Gaussian Blur...", "sigma=32");

imageCalculator("Subtract create", "FIB","FIB-1");

selectWindow("Result of FIB");

setAutoThreshold("Default dark");

//Use of fixed threshold for a given series of staining...

setThreshold(36,255);

run("Convert to Mask");

//... and discard small regions (<10 pixels) to detect real fibrosis

run("Analyze Particles...", "size=10-Infinity pixel show=Masks in_situ");

fib=titre+"_fibrosis.tif";

saveAs("Tiff", dir+fib);

selectWindow("BG");

close();

selectWindow("FIB");

close();

selectWindow("FIB-1");

close();

selectWindow("image (blue)");

close();

selectWindow("image (green)");

close();

selectWindow("image (red)");

close();

selectWindow(fib);

setAutoThreshold("Default dark");

//run("Threshold...");

run("Measure");

imageCalculator("Add", tis,fib);

setAutoThreshold("Default dark");

run("Measure");

close();

close();

fib_area=floor(getResult("Area",1));

tis_area=floor(getResult("Area",2));

selectWindow("Results");

run("Close");

print(titre);

//Print results as pixels and percentages

print("fibrosis area:",fib_area);

print("tissue area:",tis_area);

print("%fibrosis area:",floor(10000*fib_area/tis_area)/100);

print("");

**Supplemental Table 1.** Primers sequences.

**Supplemental Table 2.** Echocardiographic parameters assessed in LFD and HFD mice. LFD, low fat diet; HFD, high fat diet; HR, heart rate; IVSd, diastolic interventricular septum; IVSs, systolic interventricular septum; LVDd, diastolic left ventricular diameter; LVDs, systolic left ventricular diameter; LVPWd, diastolic left ventricular posterior wall; LVPWs, systolic left ventricular posterior wall; EF, ejection fraction; FS, fractional shortening. Results are expressed as means ± SEM (n=7). **p<0.01 compared to LFD mice.
